# Lead, Zinc and Cadmium Accumulation, and Associated Health Risks, in Maize Grown near the Kabwe Mine in Zambia in Response to Organic and Inorganic Soil Amendments

**DOI:** 10.3390/ijerph17239038

**Published:** 2020-12-04

**Authors:** Patricia N. Mwilola, Ikabongo Mukumbuta, Victor Shitumbanuma, Benson H. Chishala, Yoshitaka Uchida, Hokuto Nakata, Shouta Nakayama, Mayumi Ishizuka

**Affiliations:** 1 Department of Soil Science, School of Agricultural Sciences, University of Zambia, Lusaka P.O. Box 32379, Zambia; mwilolanalishebo@gmail.com (P.N.M.); vshitumbanuma@unza.zm (V.S.); bchishala@unza.zm (B.H.C.); 2 Faculty of Veterinary Medicine, Hokkaido University, Kita 18, Nishi 9, Kita-Ku, Sapporo 060-0809, Japan; hokuto.nakata@vetmed.hokudai.ac.jp (H.N.); shouta-nakayama@vetmed.hokudai.ac.jp (S.N.); ishizum@vetmed.hokudai.ac.jp (M.I.); 3 Research Faculty of Agriculture, Hokkaido University, Kita 9, Nishi 9, Kita-ku, Sapporo 060-8589, Japan; uchiday@chem.agr.hokudai.ac.jp

**Keywords:** legacy mining, heavy metals, soil amendments, maize, health risks

## Abstract

Health risks due to heavy metal (HM) contamination is of global concern. Despite concerns of high levels of HMs in soils near Kabwe mine in Zambia, edible crop production is common, posing potential health risks. This study assessed the potential of chicken manure (CM), triple superphosphate (TSP) and a blended fertilizer (BF; consisting of Nitrogen, Phosphorous and Potassium (NPK) fertilizer and composted chicken manure) to reduce lead (Pb), zinc (Zn) and cadmium (Cd) in soils and their accumulation in maize grown near the Kabwe mine. Maize was grown to maturity and its HM concentrations and associated health risk indices were calculated. All soil amendments decreased bioavailable soil Pb concentrations by 29–36%, but only CM decreased Zn, while the amendments increased or had no effect on Cd concentrations compared to the control. The amendments reduced Pb (>25%) and Zn concentrations (>18%) in the maize stover and grain. However, Cd concentrations in maize grain increased in the BF and TSP treatments. Bioaccumulation factors showed that Cd had the highest mobility from the soil into maize stover and grain, indicating the need for greater attention on Cd in Kabwe despite its apparently lower soil concentration compared to Pb and Zn. The hazard quotients for Pb and Cd were much greater than one, indicating a high risk of possible exposure to toxic levels by people consuming maize grain grown in this area. This study demonstrated the significant potential of manure and phosphate-based amendments to reduce Pb and Zn, and to some extent Cd, uptake in maize grain and consequently reduce associated health risks.

## 1. Introduction

Contamination of the environment due to heavy metals (HMs) from mining and other industrial activities is of major concern globally due to their effect on water and food quality and the resulting effect on human healthy [1,2,3]. Heavy metals accumulate in soils following the disposal of tailings, dust emission, through water transport or pesticide use [4,5,6,7,8]. Food crops can accumulate high amounts of HMs through soil-plant uptake, leading to food contamination and significant human health risks [8,9,10,11,12]. Food crop consumption has been reported as one of the major pathways for human exposure to HMs [11]. Furthermore, due to being non-biodegradable, HMs can accumulate in the human body even when their concentrations in the ingested food are low.

Heavy metals pose a significant effect on the health of soils, plants, animals and humans [4,8,13]. Among the HMs, lead (Pb) and cadmium (Cd) have serious adverse effects on plant growth, and human and animal health when ingested. Cd is reported to be carcinogenic and highly toxic to humans and animals [14] and leads to reduced germination and growth of plants [15]. Pb affects neurodevelopment of humans especially children [14,16] and inhibits plant growth. Unlike Pb and Cd, Zinc (Zn) is an essential plant nutrient but at elevated concentrations it has adverse effects on soil microorganisms, plants and animal health [5,15] and decreases soil quality [17].

Different strategies such as soil inversion or replacement [18], phytoremediation [19], use of raised garden beds [20], use of specific plants/crops [21] and addition of soil amendments [22,23,24], have been used to minimize accumulation of HMs in food crops and the resulting human exposure. However, phytoremediation takes long to show positive effects [25] and other methods such as soil replacement are very costly [26]. Soil amendments, such as manure and phosphate-fertilizers can be both effective and cheaper [20,26] and therefore more suited for poorer countries. Soil amendments such as manure, compost and phosphorous-based fertilizers have been proposed and shown to immobilize HMs in soil and reduce plant uptake [4,22,23,24,27,28]. Organic amendments such as animal manure immobilize HMs through chelation, formation of insoluble complexes, adsorption on charged functional groups and cation exchange [4,22,29]. Phosphate amendments immobilize HMs by formation of insoluble compounds such as pyromorphite [22,24].

In Kabwe, a town in central Zambia, mining was the main economic activity for several years but despite the closure of the mine, small-scale mining, including scavenging by local residents, is still widespread [30,31]. These activities, combined with poor environmental management have resulted in high levels of HMs such as Pb, Cd and Zn in many parts of the town [30,32]. Gardening and food crop production in the areas close to the mine is common and therefore, posing a high risk of human exposure to toxic levels of these HMs through food crop consumption. To date, there is limited information on the extent of this risk and on possible management and mitigation measures to remedy the problem [30]. Although inorganic and organic amendments, such as phosphate and manure, have shown potential to mitigate the accumulation of HMs in food crops [4,20,22,23,24], there is still lack of site-specific information in many African countries like Zambia [20,33]. In-situ field studies like this one are therefore still very crucial to fully understand the interaction between soil amendments and HMs in diverse settings and locations [20]. Furthermore, to the best of our knowledge, there haven’t been in-situ field experiments in the vicinity of the Kabwe mine to assess the extent of the human health risks through maize consumption and potential mitigation measures.

The main goals of this study were to: (i) assess the levels of accumulation of Pb, Zn and Cd in the roots, stover and grain of maize (Zea mays), a crop widely grown and consumed in the study area, and the associated health risks, based on the joint United Nations Food and Agricultural Organization (FAO) and World Health Organization (WHO) limits for human health, and (ii) evaluate the effect of phosphate-bearing organic and inorganic soil amendments: chicken manure, triple super phosphate and an NPK fertilizer mixed with chicken manure on the bioavailable concentrations of Pb, Zn and Cd in the soil and their uptake by maize. We hypothesized that although the maize could be contaminated due to proximity to the mine, the HM concentrations and the health risks of consuming the maize can be significantly reduced after applying amendments to the soils.

## 2. Materials and Methods

### 2.1. Study Site

This study was conducted on a field located within 500 m from the former Pb/Zn mine in Kabwe town, central Zambia (14°27′28.3″ S, 28°27′37″ E). Before this study, this field had been used for growing maize and other crops by local residents. The permission to use the land for this study was granted by the Kabwe municipal council.

This area receives an average of 900 mm rainfall annually with a mean annual temperature of 20.2 °C [30]. The soils at this site are classified as Chromic Haplic Lixisol [34]. The main properties of the soil and amendments are shown in Table 1.

### 2.2. Experimental Design

This study examined the effects of raw chicken manure (CM), triple super phosphate (TSP), and blended fertilizer (BF)—a mixture of inorganic NPK fertilizer (10:20:10) and chicken manure on Pb, Zn and Cd immobilization in the soil and uptake in maize. These amendments were chosen in part due to ease of use, high availability in the study site and relatively low cost. Chicken manure was used its raw form as this is the common practice among gardeners in Zambia. Including the un-amended control (CT), there were a total of 16 plots each 4 × 4 m in size. Each of the four treatments was replicated four times and arranged in a Latin square design with a space of 0.5 m left between the plots. The field was ploughed to ~20 cm depth before application of the amendments. The amount of each amendment applied was aimed at supplying enough phosphate (PO_4_) to immobilize the Pb within the top 20 cm soil by converting it to pyromorphite (Pb_5_(PO_4_)_3_C/) at 5:3 Pb:phosphorous (P) ratio [4,35]. Although the soil contains different HMs, the amendments were applied based on the concentration of Pb, the metal of highest concern in the area [30]. The P contents in CM, BF and TSP were 1.2%, 20% and 46% respectively, resulting in the application of 82 kg CM, 5 kg BF and 3.2 kg TSP per plot. The applied amendments were equivalent to 50 ton, 6 ton and 0.9 ton ha^−1^ CM, BF and TSP respectively. These application rates of the amendments were similar to those used in other studies [4,23]. Nitrogen equal to that applied in the BF treatment (300 g plot^−1^, 187 kg N ha^−1^) was applied in the TSP and CT plots. The amendments were incorporated into the soil to the rooting depth of 20 cm [36,37].

The maize was planted at 20 and 40 cm intra- and inter-row spacing. At physiologic maturity, about 125 days after planting, maize plants were harvested and separated into roots, stover and grain. To determine total maize biomass production, maize plants in each plot were weighed and then 10 plants selected for drying used for determination of the moisture content and HM concentration. After drying (at 70 °C) the roots, stover and grains were ground and homogenized. The HM concentrations in the different plant parts were extracted in 1 M HNO_3_ after dry ashing [38] and analyzed using a Flame Atomic Adsorption Spectrophotometer (FAAS; AA-6300, Shimadzu, Kyoto, Japan). The FAAS was run using acetylene gas and air as an oxidant at 2.5 mL min^−1^. The detection limit for the FAAS was 0.01, 0.002 and 0.0004 mg L^−1^ for Pb, Zn and Cd respectively.

### 2.3. Determination of Total and Bioavailable Pb, Zn and Cd in Soils

Soil samples (0–20 cm depth) were collected prior to starting the experiment at 10 points within a 50 × 50 m field, and in each plot at the end of the experiment. The soils were air-dried, homogenized and then passed through a 2 mm sieve. The soils were analyzed for total Pb, Zn and Cd contents extracted using aqua-regia solution (a mixture of nitric acid and hydrochloric acid in 1:3 ratio), and potentially-plant available Pb, Zn and Cd concentrations extracted using diethylentriaminopentanacetic acid triethanolamine (DTPA-TEA) solution buffered at pH 7.3 according to Lindsay and Norvell [39]. The DTPA-TEA extractable HMs are considered as bioavailable for plant uptake in this study [40]. Both the total and potentially-plant available soil Pb, Zn and Cd concentrations were determined using a FAAS.

For quality control, standard reference materials (SRM) 1573a (dried tomato leaves) and 2710a (Montana soil) were used. Additionally, all reagents used for both plant and soil extractions were analytical grade. For every set of sample analysis, a blank, with only the extracting reagent, was run during both the digestion and analysis throughout the experiment, and additionally, all containers were thoroughly washed, immersed in diluted HNO_3_ for 24 h and subsequently rinsed with deionized water before use. Standard solutions were used to precondition the FAAS during each analysis. All plant and soil analyses were replicated four times.

### 2.4. Determination of Bioaccumulation Factors for Pb, Zn and Cd in Maize Stover and Grain

The bioaccumulation factor (BAF) is an important index that shows the transfer of HMs and other hazardous materials from the soil into plants [8]. In this study, the BAFs were determined separately for transfer of HMs from soil into the maize stover and from the soil into the maize grain using Equation (1).

BAF = HM concentration in stover (or grain)/Soil HM concentration(1)

### 2.5. Pb and Cd Dietary Intake and Health Risk Assessment

The Joint United Nations Food and Agricultural Organization (FAO)/WHO Codex Alimentarius Commission (2018) [41] maximum tolerable intake limits were used as critical values to check the extent to which maize grown in soils near the Kabwe mine pose a risk to the local people. The hazard quotient (HQ) was used to assess the potential risk to human health resulting from maize consumption and was calculated as follows:

HQ = EI/TI(2)

EI = HM concentration in grain ∗ Df ∗ Y/HBW(3)

where: EI is the estimated metal intake (weekly for Pb and monthly for Cd) through maize grain consumption; TI represents the safe level of metal exposure through food crop consumption (weekly for Pb and monthly for Cd) [41]; Df is the average daily consumption of maize grain for an adult in Zambia; HBW is the average human body weight of an adult; Y is the number of days in a week or month.

In this study, 350 g was used as the Df [42], 70 kg as HBW [43] and 0.025 mg kg^−1^ HBW as the monthly Cd and weekly Pb TI values [41].

HQ values greater than 1 indicate significant risk to human health, while values less than 1 are considered safer for human health [10,40].

The TI value for Zn has not been established yet [41,43] and therefore EI and HQ values were not calculated for Zn in this study.

### 2.6. Data Analysis

All statistical analyses were performed using STATA 13 (Stata Corporation, College Station, TX, USA). The effect of soil amendments on bioavailable Pb, Zn and Cd concentrations in soils, and Pb, Zn and Cd concentrations in maize roots, stover and grain were evaluated using one-way ANOVA. Differences among the amendments were tested using Tukey’s significance test. Pearson’s correlation analysis was used to establish relationships among the bioavailable Pb, Zn and Cd concentrations in soils, Pb, Zn and Cd concentrations in maize roots, stover and grain with maize biomass

## 3. Results

### 3.1. Effect of Soil Amendments on Potentially-Plant Available Pb, Zn and Cd and Maize Biomass Production

**Figure 1:**
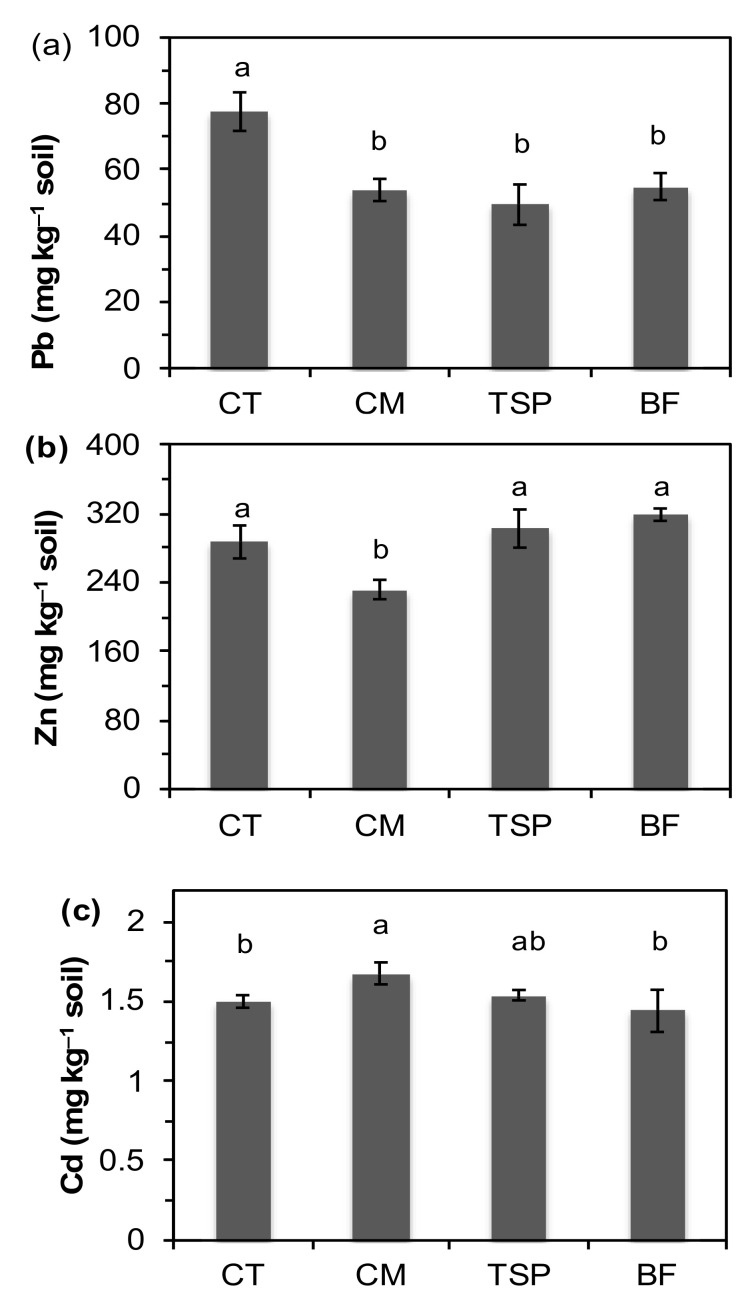
Concentrations of bioavailable: (**a**) lead (Pb); (**b**) zinc (Zn); (**c**) cadmium (Cd) in the soil at the end of the experiment. CT is the control, CM is chicken manure, TSP is triple superphosphate, BF is blended fertilizer (a mixture of NPK fertilizer and chicken manure). Bars with different letters are significantly different (*p* < 0.05).

The DTPA-TEA extractable Pb concentrations in the soil decreased by 30%, 36% and 29% in the CM, TSP and BF treatments respectively, compared to the unamended control plots (Figure 1). In contrast to results for Pb, TSP and BF did not decrease DTPA-TEA extractable Zn or Cd compared to the un-amended soil (Figure 1). Chicken manure on the other hand decreased concentrations of DTPA-TEA extractable Zn by 19% but increased those of Cd by 10% compared to the control.

Relative to the control treatment, the maize biomass production significantly increased in the CM and BF amended plots (Table 2). Maize biomass in the TSP plot was higher than that in the control although not statistically significant.

### 3.2. Effect Soil Amendments on Pb, Zn and Cd Accumulation in Maize

**Figure 2:**
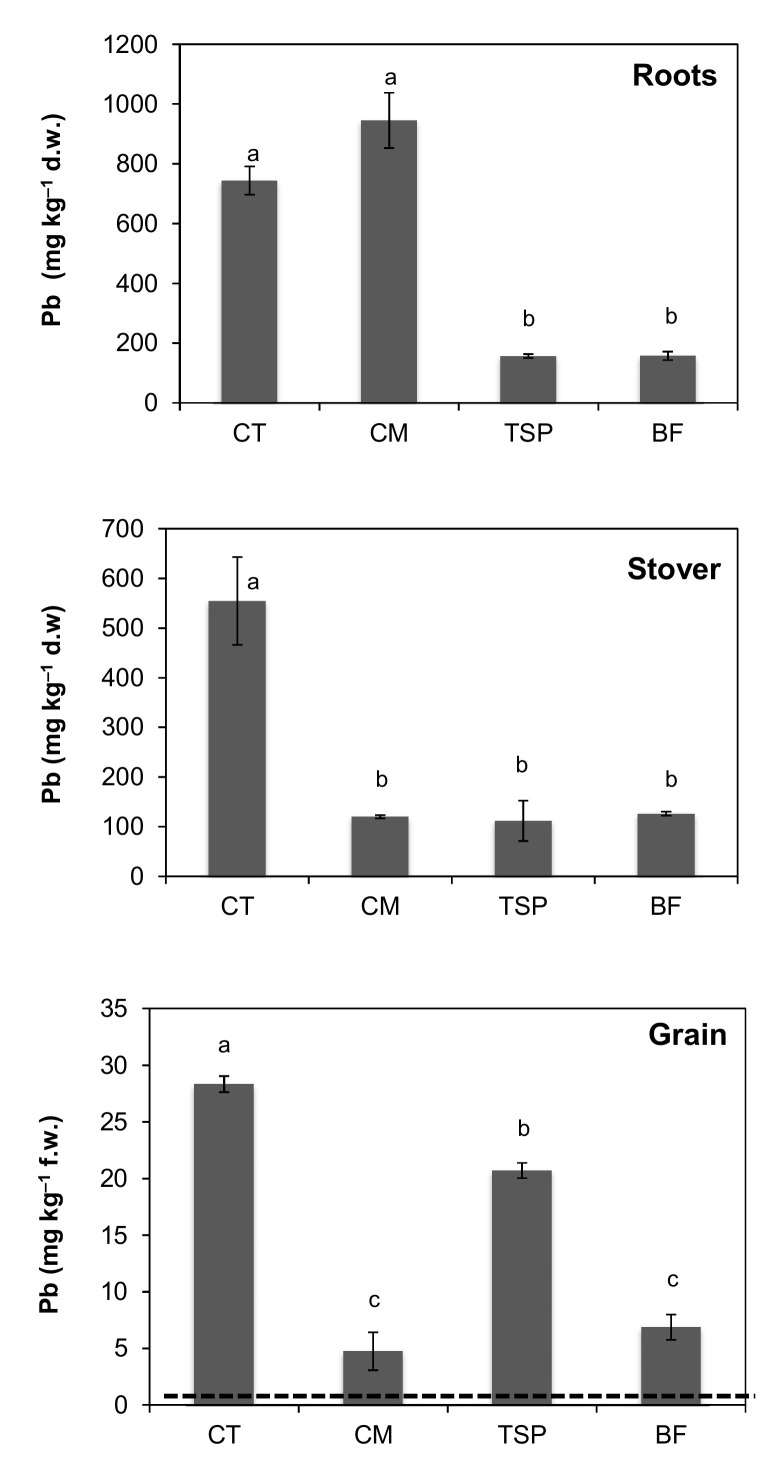
Concentrations of lead (Pb) in maize roots, stover and grain in plots amended with chicken manure (CM), triple superphosphate (TSP) and blended fertilizer (BF; a mixture of NPK fertilizer and chicken manure) compared to the control (CT). The horizontal broken line indicates the FAO/WHO maximum allowable limit of 0.2 mg Pb kg^−1^ maize grain for human health. Concentrations in roots and stover are on dry matter weight (d.w) basis, but on fresh weight (f.w) basis in grain. Bars with different letters are significantly different (*p* < 0.05).

Lead concentrations were highest in roots and lowest in the maize grain (Figure 2). Application of TSP and BF decreased Pb concentration in roots by more than 75% with root Pb concentration averaging 156.2 and 157.1 mg kg^−1^ in TSP and BF treatments compared to 743.3 mg kg^−1^ in the control plot. Manure application on the other hand did not decrease root Pb concentrations and instead resulted in a slight increase. Unlike the concentrations of Pb in roots, all three soil amendments significantly (*p* < 0.01) reduced Pb concentrations in the maize stover from 554 mg kg^−1^ to below 130 mg kg^−1^, on average, representing a reduction of more than 75% (Figure 2). In the maize grain, Pb concentration decreased by 27%, 76% and 83% in the treatments TSP, BF and CM, respectively, compared to the control (Figure 2). The maize grain Pb concentrations ranged from 27.3–29.0, 3.1–5.3, 20.6–21.6 and 5.8–8.4 mg kg^−1^ in the un-amended control, CM, TSP and BF treatments, respectively. The Pb concentrations in the maize grain, from all treatments, were higher than the joint FAO/WHO maximum permissible limit of 0.2 mg kg^−1^ for human health safety.

**Figure 3:**
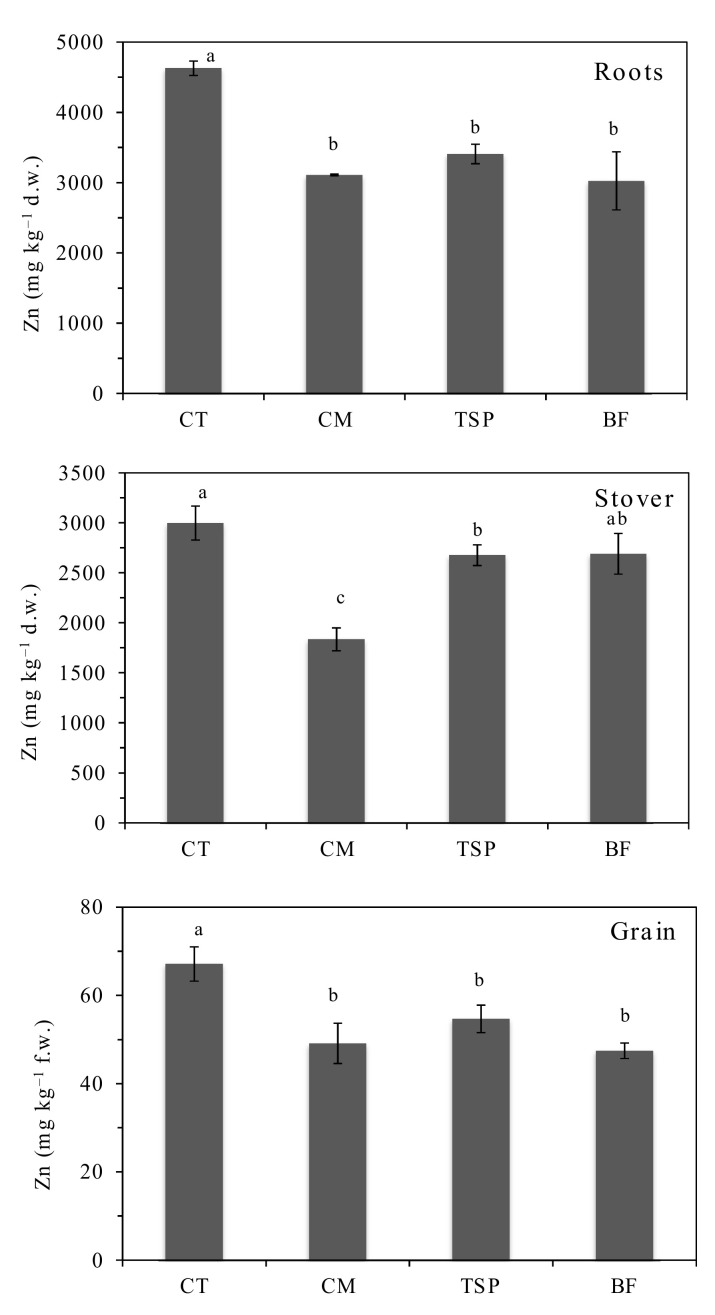
Concentrations of zinc (Zn), in maize roots, stover and grain in plots amended with chicken manure (CM), triple superphosphate (TSP) and blended fertilizer (BF; a mixture of NPK fertilizer and chicken manure) compared to the control (CT). Concentrations in roots and stover are on dry matter weight (d.w) basis, but on fresh weight (f.w) basis in grain. Bars with different letters are significantly different (*p* < 0.05).

Zinc concentrations in different maize parts generally decreased with soil amendment application (Figure 3). In the roots, the Zn concentration in the amended soils ranged from 3100–3400 mg kg^−1^ compared to an average of over 4600 mg kg^−1^ in the un-amended soils, representing a 26–33% decrease (Figure 3). Chicken manure and TSP significantly (*p* < 0.01) decreased Zn concentration in the stover. A significant reduction of 18–29% in maize grain Zn concentration was observed in all three soil amendments compared to the control, but there was no significant difference among the three amendments. The maize grain Zn concentrations ranged from 62.4–71.9, 45.2–55.3, 50.9–58.5 and 45.7–49.9 mg kg^−1^ in the un-amended control, CM, TSP and BF treatments, respectively.

**Figure 4:**
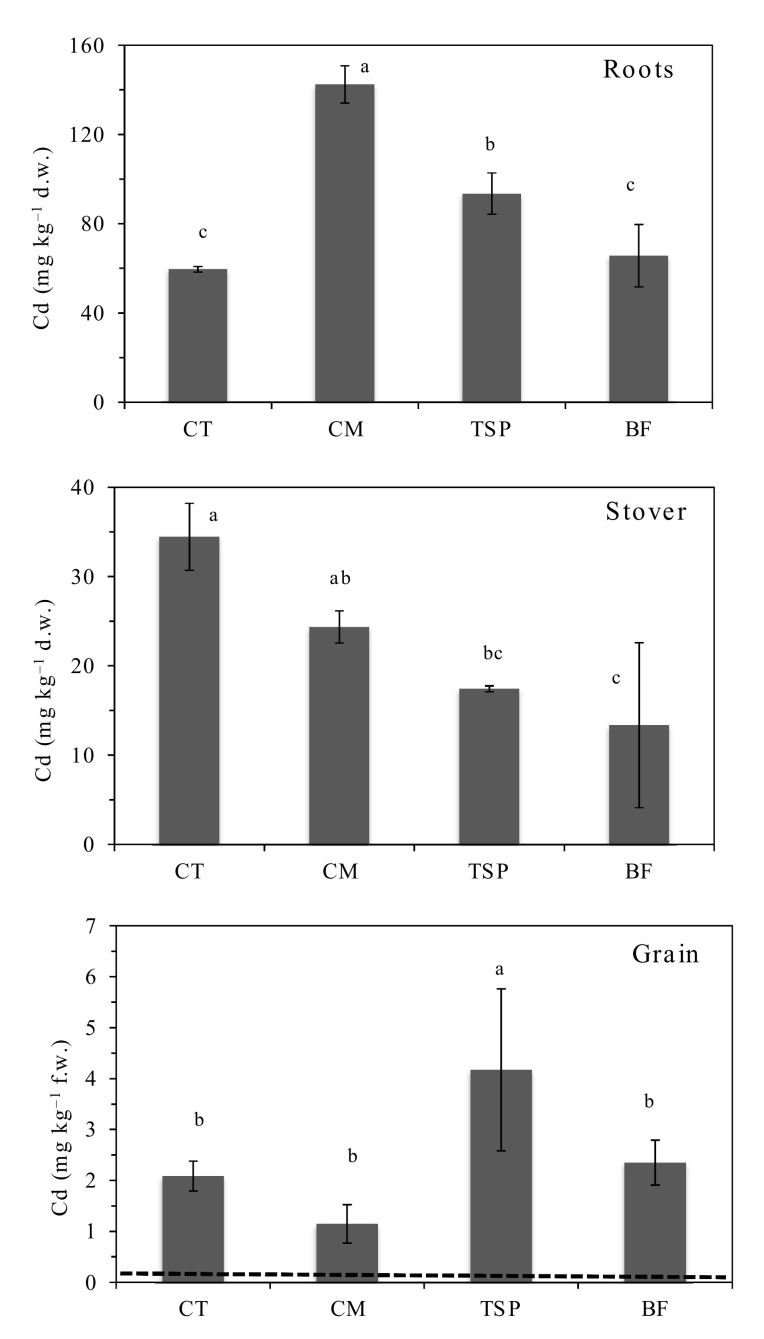
Concentrations of cadmium (Cd), in maize roots, stover and grain in plots amended with chicken manure (CM), triple superphosphate (TSP) and blended fertilizer (BF; a mixture of NPK and chicken manure). The horizontal broken line indicates the FAO/WHO maximum allowable limit of 0.1 mg Cd kg^−1^ maize grain for human health. Concentrations in roots and stover are on dry matter weight (d.w) basis, but on fresh weight (f.w) basis in grain. Bars with different letters are significantly different (*p* < 0.05).

Chicken manure and TSP significantly increased Cd concentrations in the roots, while all the soil amendments decreased Cd concentrations in the stover compared to the control (Figure 4). Cadmium concentrations in maize grain were significantly higher (*p* < 0.05) in TSP amended plots than other amendments including the control. The maize grain Cd concentrations ranged from 1.7–2.4, 0.8–1.5, 2.4–5.6 and 1.8–2.9 mg kg^−1^ in the un-amended control, CM, TSP and BF treatments, respectively. Cadmium accumulation in maize grain in CM, BF and control was not significantly different, but the mean maize grain Cd concentration in CM was lower than that in the control. Cadmium concentrations in the grain were 20, 11, 41 and 23 times higher than the joint FAO/WHO maximum limit of 0.1 mg kg^−1^ in the control, CM, TSP and BF treatments respectively.

Lead concentrations in maize grain were positively correlated with Pb concentrations in soil and stover, but not with Pb concentrations in roots (Table 3). There was no correlation between Zn concentrations in maize grain and Zn concentrations in soil, but Zn concentrations in the roots had positive correlations with Zn in the stover. The biomass production was negatively correlated with Zn and Pb concentrations in the stover and grain, but only weakly, and not statistically significant, correlated with HM concentrations in the soil (Table 3).

### 3.3. Effect of Soil Amendments on Bioaccumulation Factors of Pb, Zn and Cd

**Figure 5:**
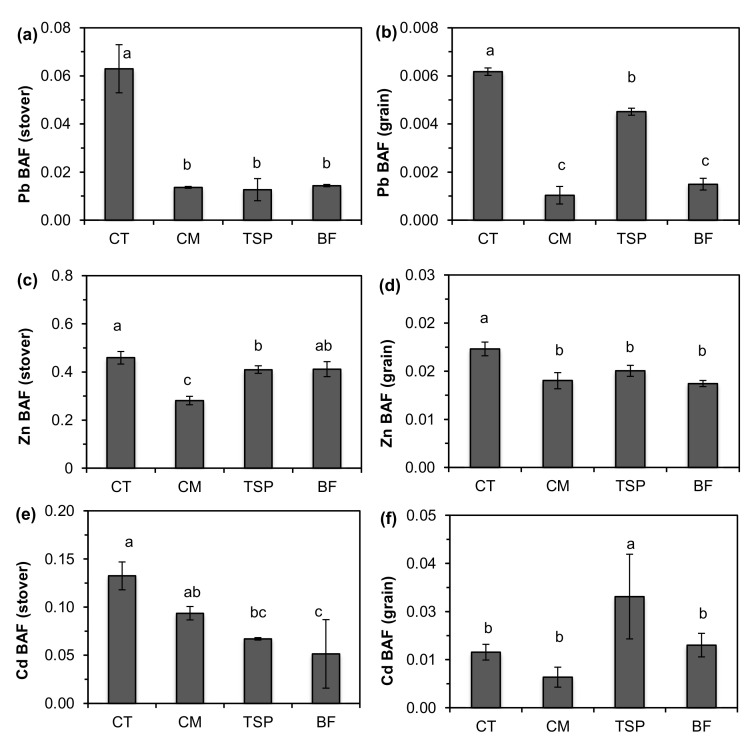
Bioaccumulation values for (**a**,**b**) Pb; (**c**,**d**) Zn; (**e**,**f**) Cd of maize stover and grain in plots amended with chicken manure (CM), triple superphosphate (TSP) and blended fertilizer (BF; a mixture of NPK fertilizer and chicken manure) and the control (CT). Bars with different letters are significantly different (*p* < 0.05).

The bioaccumulation factors (BAF) of the stover and grain, which indicate the transfer of HMs from soil into above-ground plant parts, are shown in Figure 5. There was a 6-fold reduction in the BAF_Pb_ of maize stover with the application of soil amendments. Chicken manure and BF resulted in 8- and 5-fold reductions in the BAF_Pb_ in the maize grain, while TSP had a 19% reduction compared to the control. Soil amendments significantly decreased the BAF_Zn_ of the stover and grain, except for TSP in stover (Figure 5). The soil amendments significantly decreased the BAF_Cd_ in maize stover, but TSP increased the BAF_Cd_ in the maize grain. Overall, the BAF values were in the order Zn ≥ Cd > Pb.

### 3.4. Estimated Dietary Intake and Hazard Quotient Assessment of Pb and Cd

A preliminary hazard assessment for human Pb and Cd ingestion through consumption of maize grain is shown in Table 4. The assessment was based on the assumption that daily intake of maize grain for an average adult Zambian weighing 70 kg is 350 g day^−1^ [42]. The estimated weekly Pb ingestion from maize consumption was highest in the un-amended control soils and lowest from soils amended with chicken manure. Although higher than the joint FAO/WHO maximum tolerable Pb weekly intake of 0.025 mg kg^−1^ human body weight (HBW), estimated weekly Pb ingestion decreased by 83%, 27% and 75% with application of CM, TSP and BF, respectively.

The estimated monthly Cd ingestion from maize grain consumption increased by 100% with the application of TSP compared to the control. Chicken manure reduced the estimated monthly Cd ingestion by 40% compared to the control. This difference was however not statistically significant. The monthly Cd ingestion in all treatments was higher than the joint FAO/WHO maximum tolerable limit of 0.025 mg Cd kg^−1^ HBW.

The HQ values for both Pb and Cd in all treatments were much greater than 1, indicating that the maize grain grown in these soils was not safe for human consumption (Table 4). However, the HQ values for Pb were significantly reduced in plots with CM and BF. Plots with TSP had the highest HQ values for Cd.

## 4. Discussion

### 4.1. Effects of Soil Amendments on Heavy Metal Bioavailability and Plant Uptake

The reduction in bioavailable HMs in the soil after application of manure and P based amendments has been reported by other studies [4,22,23,24]. Contrary to other studies (e.g., Putwattana et al., 2015), no significant reduction in bioavailable Cd and Zn concentrations were observed after applying inorganic P amendments in this study. This finding is in agreement with that of a laboratory study by Bohdan et al. (2019) who reported that inorganic P amendment decreased concentrations of bioavailable Pb but not Zn, and that humate application did not affect bioavailable Zn and Cd in Kabwe soils. Unlike the findings of Bohdan et al. (2019), in this study bioavailable (DTPA-TEA extractable) Cd did not decrease after applying inorganic P amendments. Results of this study suggest that a single amendment was unlikely to decrease the bioavailability of all the three HMs of concern in Kabwe, and therefore future studies should look at different combinations of these amendments.

The decreased uptake of Pb and Zn in the maize in the amended soils was likely due to their immobilization in the soil through the formation of insoluble precipitates or complexes [23,24], or due to fixation to organic matter functional groups [9], or ion exchange due to increased soil surface charge [23] following application of organic amendments like CM and BF. Despite increasing Pb concentration in plant roots, the phosphate and organic amendments reduced Pb translocation from the roots to shoots. This could be due to the formation of pyromorphite-like minerals on the root membrane surface [24] or to inhibited metal transfer within the plant [23].

The reduction of HM concentrations in maize stover and grain in plots amended with CM, TSP and BF (Figure 2, Figure 3 and Figure 4) could also have been due to the “growth dilution effect” [4,23] following higher biomass production compared to the control. In this study, the maize biomass yield in plots amended with CM and BF was 25 times higher, while the biomass in the plots with TSP was 6 times higher than that in the control (Table 2). Increased plant growth, which likely resulted from the increased availability of plant nutrients in the soil and decreased plant stress due to reduced soil HM bioavailability, could have reduced the concentration of the HMs in the aboveground plant parts. The observed higher Cd concentrations in the maize grain from plots with TSP and BF amended soils than that in the control (Figure 4) was likely due to the reported increased solubility of Cd in the presence of phosphate compounds [22]. Some Cd-phosphate complexes are reported to have relatively high solubility in the presence of high phosphate [22], making the Cd more mobile.

Other studies have suggested that inorganic phosphate fertilizers increase Cd uptake in plants due to the introduction Cd to the soil by fertilizers [14,22]. Studies have shown that inorganic phosphate fertilizers tend to increase Cd concentrations in soils [14,22], because some rock phosphates used to produce phosphate fertilizers contain high levels of Cd. It is unlikely that increased Cd the maize grain could have resulted from the fertilizers because the Cd concentrations in all three amendments were below the detection limit in the current study. However, this cannot be ruled out completely.

The soil-to-plant bioaccumulation factors of all three HMs were higher for maize stover than for grain, indicating a possible defense mechanism against the translocation of excess HMs into grains [40]. The general trend was that Zn showed the highest bioaccumulation factors while Pb had the lowest. However, in the maize grain, the bioaccumulation factors for Cd were either of similar magnitude or even higher than those for Zn. This suggests that once in the plant, Cd had a higher ability for translocation than both Zn and Cd. High Cd mobility in plants has been reported by Bi et al. [10].

### 4.2. Health Risk Assessment

The concentrations of Pb and Cd in the maize grain (Figure 2 and Figure 4) were higher than the Joint FAO/WHO Codex Alimentarius Commission [41] limits for food safety. In the un-amended soil, the Pb concentrations in the maize grain were more than 100 times higher than the joint FAO/WHO maximum permissible limit of 0.2 mg kg^−1^ for health safety but decreased significantly (only 24 times higher) in the chicken manure amended plots. High Pb and Cd concentrations in maize in Kabwe was previously reported by Nakata et al. [44]. These high concentrations resulted in high estimated intake (EI) values as well as hazard quotients (HQ). The HQ values were higher than 1 (Table 4), indicating significant human health risk. Higher HQ values near former mining sites have been reported by other studies [5,10,40]. Despite relatively low Cd concentrations in the soils, Cd concentrations in the maize grain were high. This could be due to the reported high mobility and transfer of Cd from soil into plants [5].

Maize is widely grown and consumed in areas surrounding the old Kabwe mine and throughout Zambia. Results of this study have shown that the concentrations of Pb and Cd in maize grain grown on soils near the former Kabwe Pb/Zn mine were higher than the maximum tolerable limits for food safety and human health. It is therefore clear, from this study, that the risk of human exposure to toxic levels of Pb and Cd by consuming maize grown in areas surrounding the mine is very high. This study also demonstrated that there is a high potential to reduce this risk by applying amendments such as chicken manure and phosphate-based fertilizers to these soils. Because this study was conducted at a site within 500 m from the old Pb-Zn mine with very high HM concentrations [30], it is possible that on sites much further away from the mine, the application of amendments tested in this study could potentially decrease HM concentrations to below the maximum tolerable limits and further reduce the hazard quotients. Additionally, these results are useful to various parts of Zambia and the world at large, where contamination exists either from mining or other sources such as pesticide use [4]. Compared to phosphate fertilizer, chicken manure is relatively widely available and cheaper for most local residents in low-income mining townships in Zambia where backyard gardening is widely practiced. Hence using chicken manure could not only reduce the HM accumulation in edible crops but also to increase productivity and is a potentially low-cost management option.

The observed high health risk of Cd requires that serious attention be paid to the problem of Cd pollution, which has currently been overshadowed by that of Pb, owing to the higher concentrations of Pb than Cd in Kabwe soils. Therefore, while Pb receives its due attention by researchers, health and civic authorities and the public at large, the problems posed by Cd in the environment should equally be of concern in Kabwe.

## 5. Conclusions

All three soil amendments (chicken manure, triple superphosphate and NPK fertilizer mixed with chicken manure) decreased bioavailable soil Pb concentrations, but only chicken manure decreased Zn and Cd in soil compared to the control. The soil amendments reduced Pb and Zn concentrations in the maize stover and grain. Amendments containing inorganic phosphate increased Cd concentrations in maize grain. The health risk assessment reviewed that Pb and Cd concentrations in maize, and the resulting estimated weekly and monthly metal intakes exceeded the FAO/WHO limits. The hazard quotients for Pb and Cd indicated that there is a high health risk to people who consume maize grain grown on the soils in the study area. Results of this study have demonstrated that the use of chicken manure and phosphate-based soil amendments have significant potential to reduce the concentrations of Pb, Zn and Cd in maize grown in the study area and consequently reduce related health risks associated with the consumption of the maize.

## Figures and Tables

**Table 1:** Soil and amendment characteristics.

Parameter	Soil	Manure	BF	TSP
**Total Pb (mg kg**^**−1**^)	8810 ± 310	-	-	-
**Total Zn (mg kg**^**−1**^)	1102 ± 203	-	-	-
**Total Cd (mg kg**^**−1**^)	260 ± 17	-	-	-
**Total P**	21.0 mg kg^−1^	1.2%	20%	46%
**Total carbon (%)**	3.7			
**Total nitrogen (%)**	0.28	3.0	10	0
**pH**	5.7			
**CEC (cmol + kg^−1^)**	5.2			

CEC is cation exchange capcity; P is phosphorous; BF is blended fertilizer (a mixture of NPK fertilizer and chicken manure; TSP is triple super phosphate.

**Table 2:** Mean maize biomass yield (±standard error) for different treatments.

	CT	CM	TSP	BF
**Biomass Yield** (**kg plot^−1^**)	0.9 ± 0.2 ^b^	22.7 ± 2.2 ^a^	5.2 ± 0.4 ^b^	22.5 ± 3.7 ^a^
**Biomass Yield** (**kg ha^−1^**)	1164 ± 310 ^b^	28,438 ± 2830 ^a^	6578 ± 529 ^b^	28,092 ± 4726 ^a^

CT is the control, CM is chicken manure, TSP is triple superphosphate, BF is blended fertilizer (mixture of NPK fertilizer and chicken manure) treatments. Values with different superscripts within a row indicate significantly different treatment means at *p* < 0.05.

**Table 3:** Correlations between metal concentrations in soil and plant parts and biomass yield.

	**Pb Grain**	**Yield**
Pb soil	0.59 *	−0.45
Pb roots	−0.03 ^ns^	0.06 ^ns^
Pb stover	0.74 **	−0.61 *
Pb grain	1	−0.92 ***
	**Zn Grain**	**Yield**
Zn soil	0.04 ^ns^	−0.26 ^ns^
Zn roots	0.86 ***	−0.66 **
Zn stover	0.60 *	−0.66 **
Zn grain	1	−0.75 ***
	**Cd Grain**	**Yield**
Cd soil	−0.30 ^ns^	0.18 ^ns^
Cd roots	−0.28 ^ns^	0.38 ^ns^
Cd stover	−0.33 ^ns^	−0.41 ^ns^
Cd grain	1	−0.47 ^ns^

* *p* <0.05; ** *p* < 0.01; *** *p* < 0.001, ns not significant.

**Table 4:** Estimated weekly Pb and monthly Cd intakes (EI) and hazard quotient (HQ) for raw uncooked maize grain grown in plots with different soil amendments compared to joint United Nations Food and Agricultural Organization (FAO)/WHO food tolerable intake limits [41].

	CT ^†^	CM	TSP	BF	FAO/WHO Limit
EI_Pb_ (mg/kg HBW/week)	0.99 ± 0.02 ^a^	0.16 ± 0.05 ^b^	0.72 ± 0.02 ^a^	0.24 ± 0.04 ^b^	0.025
EI_Cd_ (mg/kg HBW/month)	0.31 ± 0.04 ^b^	0.17 ± 0.05 ^b^	0.62 ± 0.23 ^a^	0.35 ± 0.06 ^b^	0.025
HQ_Pb_	39.6 ± 1.0 ^a^	6.6 ± 2.3 ^b^	28.9 ± 0.9 ^a^	9.6 ± 1.5 ^b^	
HQ_Cd_	12.5 ± 1.7 ^b^	6.8 ± 2.3 ^b^	25.0 ± 9.5 ^a^	14.1 ± 2.6 ^b^	

^†^ CT is the control, CM is chicken manure, TSP is triple superphosphate, BF is blended fertilizer (mixture of NPK fertilizer and chicken manure) treatments. Means with different superscripts within each row are significantly different at *p* < 0.05.
